# High-Fat Diet with Acyl-Ghrelin Treatment Leads to Weight Gain with Low Inflammation, High Oxidative Capacity and Normal Triglycerides in Rat Muscle

**DOI:** 10.1371/journal.pone.0026224

**Published:** 2011-10-19

**Authors:** Rocco Barazzoni, Michela Zanetti, Annamaria Semolic, Maria Rosa Cattin, Alessia Pirulli, Luigi Cattin, Gianfranco Guarnieri

**Affiliations:** Clinica Medica - Department of Medical, Surgical and Health Sciences, University of Trieste, Trieste, Italy; Mayo Clinic, United States of America

## Abstract

Obesity is associated with muscle lipid accumulation. Experimental models suggest that inflammatory cytokines, low mitochondrial oxidative capacity and paradoxically high insulin signaling activation favor this alteration. The gastric orexigenic hormone acylated ghrelin (A-Ghr) has antiinflammatory effects *in vitro* and it lowers muscle triglycerides while modulating mitochondrial oxidative capacity in lean rodents. We tested the hypothesis that A-Ghr treatment in high-fat feeding results in a model of weight gain characterized by low muscle inflammation and triglycerides with high muscle mitochondrial oxidative capacity. A-Ghr at a non-orexigenic dose (HFG: twice-daily 200-µg s.c.) or saline (HF) were administered for 4 days to rats fed a high-fat diet for one month. Compared to lean control (C) HF had higher body weight and plasma free fatty acids (FFA), and HFG partially prevented FFA elevation (P<0.05). HFG also had the lowest muscle inflammation (nuclear NFkB, tissue TNF-alpha) with mitochondrial enzyme activities higher than C (P<0.05 vs C, P = NS vs HF). Under these conditions HFG prevented the HF-associated muscle triglyceride accumulation (P<0.05). The above effects were independent of changes in redox state (total-oxidized glutathione, glutathione peroxidase activity) and were not associated with changes in phosphorylation of AKT and selected AKT targets. Ghrelin administration following high-fat feeding results in a novel model of weight gain with low inflammation, high mitochondrial enzyme activities and normalized triglycerides in skeletal muscle. These effects are independent of changes in tissue redox state and insulin signaling, and they suggest a potential positive metabolic impact of ghrelin in fat-induced obesity.

## Introduction

Obesity may be characterized by lipid accumulation in skeletal muscle, and this alteration likely contributes to long-term metabolic complications [1]. Experimental models suggest that inflammatory cytokines, changes in muscle mitochondrial function and paradoxical enhancement of insulin signaling at the AKT level contribute to increase tissue lipid deposition in the presence of weight gain and high lipid availability [1]–[3]. Pro-oxidant changes in redox state may further contribute to inflammation and altered mitochondrial function, and they are commonly associated with muscle lipid accumulation [4], [5].

Ghrelin is a gastric hormone with orexigenic and adipogenic effects that may favor weight and fat gain in vivo [6], [7]. Acylated ghrelin (A-Ghr) has been however reported to lower muscle triglyceride content in healthy and uremic lean rodents, associated with enhanced skeletal muscle mitochondrial oxidative capacity [8], [9]. Antiinflammatory and antioxidant effects of A-Ghr have been also demonstrated in vitro [10]–[12]. The impact of A-Ghr administration on muscle redox state, inflammatory mediators, mitochondrial oxidative capacity and triglyceride content following diet-induced weight gain remains however undetermined.

In the current study we therefore administered A-Ghr for four days at a non-orexigenic dose in a rodent model of high-fat diet-induced obesity. We hypothesized that A-Ghr administration results in a model of weight gain characterized by low muscle oxidative stress and inflammation, high muscle mitochondrial oxidative capacity and low tissue triglycerides. The potential association between muscle triglyceride changes and altered muscle insulin signaling at the AKT level was also investigated, since AKT activation under non-stimulated conditions has been paradoxically reported to contribute to muscle lipid accumulation during high-fat feeding [2], and tissue-specific insulin-sensitizing effects of ghrelin have been shown in non-obese experimental models [13].

## Results

### Body weight, plasma metabolic profile (Table 1)

**Table 1:** Initial body weight (BW), body weight at the end of the one-month dietary treatment (before start of ghrelin or saline injection treatments), body weight changes before start of ghrelin or saline treatments, body weight changes during 4-day ghrelin or saline treatments, total 4-day caloric intake during 4-day ghrelin or saline treatment, retroperitoneal and epidydimal fat pad weights, plasma insulin, glucose and free fatty acids (FFA) concentrations in the three experimental groups.

	Control	HF	HFG
Initial BW (g)	295±7^a^	297±9^a^	294±9^a^
BW 1-mo (g)	378±7^a^	412±12^b^	408±8^b^
BW changes 1-mo (g)	84±8^a^	115±7^b^	112±4^b^
BW changes 4-day (g)	8±1^a^	9±2^a^	15±1.5^b^
Caloric intake 4-day (cal)	505±13^a^	631±11^b^	639±12^b^
Fat – Retroperitoneal (g)	5.7±0.4^a^	9.4±0.7^b^	9.4±0.5^b^
Fat – Epidydimal (g)	5.3±0.4^a^	9.2±0.5^b^	8.4±0.5^b^
Insulin (ng/ml)	6.8±1.4^a^	5.9±0.8^a^	7.2±1^a^
Glucose (mg/dl)	107±3^a^	118±4^b^	114±3^ab^
FFA (mmol/l)	0.21±0.026^a^	0.59±0.062^b^	0.39±0.047^c^

Data are Mean±SE. Different letters denote statistically significant differences: P<0.05 by ANOVA and post-hoc tests.

Initial body weight was comparable in the three experimental groups, while final body weight and the weight of epidydimal and retroperitoneal fat were higher in HF compared to control animals. HFG had food intake and final body weight comparable to HF. Weight gain during the four-day ghrelin treatment was however moderately higher in HFG compared to HF animals, although this alteration was not associated with higher caloric intake. Final weights of the epidydimal and retroperitoneal fat pads were also comparable in HF and HFG groups. Blood glucose was higher while plasma insulin was similar in HF and control animals. In HFG, both blood glucose and plasma insulin were comparable to control and HF groups, while plasma free fatty acids were markedly lower in HFG than in the HF group.

### Muscle inflammation, mitochondrial enzyme activities and triglycerides (Figure 1–2)

**Figure 1:**
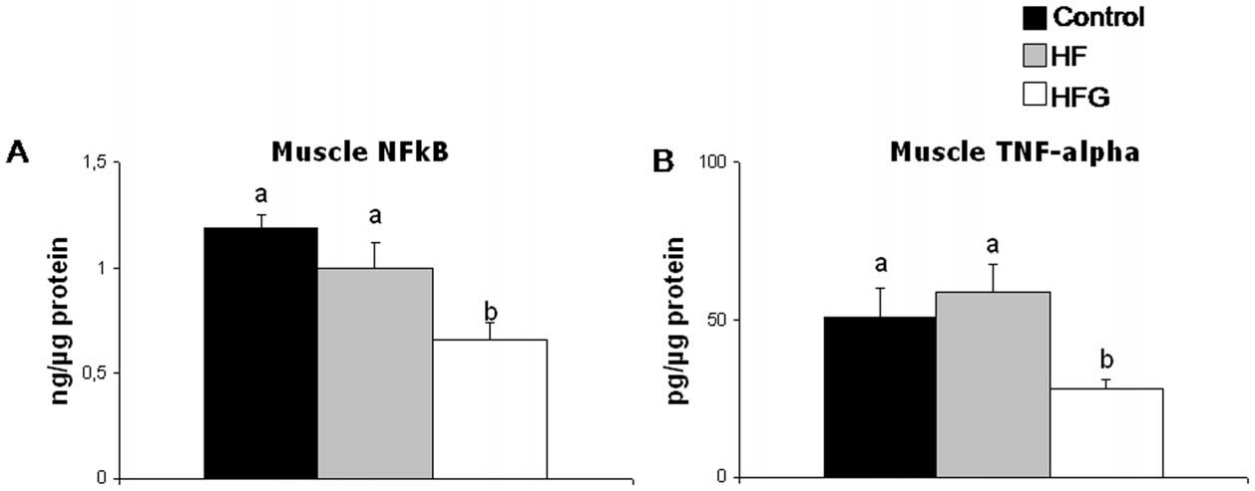
Acylated ghrelin during HF diet lowers gastrocnemius muscle proinflammatory molecules. Effects of one-month high-fat feeding without (HF) or with (HFG) 4-day acylated ghrelin treatment on gastrocnemius muscle levels of nuclear p65 NFkB subunit (a) and TNF-alpha protein (b). Different letters denote statistically significant differences (P<0.05 ANOVA and post-hoc test).

**Figure 2:**
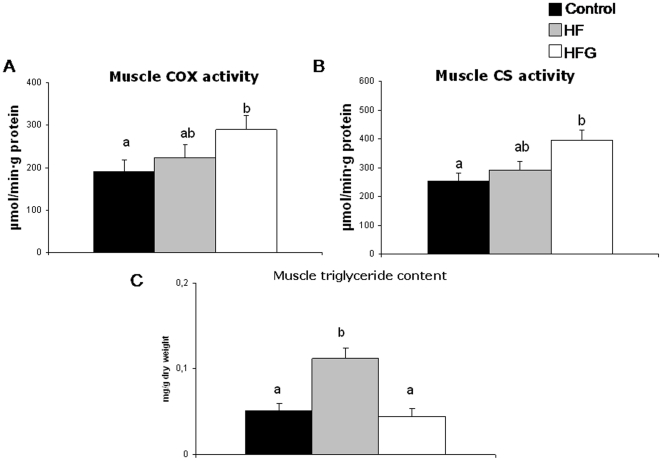
Acylated ghrelin with HF diet enhance gastrocnemius muscle mitochondrial enzyme activities. Effects of one-month high-fat feeding without (HF) or with (HFG) 4-day acylated ghrelin treatment on gastrocnemius muscle activities of mitochondrial cytochrome c oxidase (COX) (a) and citrate synthase (CS) (b) and on muscle triglyceride content (c). Different letters denote statistically significant differences (P<0.05 ANOVA and post-hoc test).

A-Ghr treatment lead to lower levels of nuclear p65 NFkB and tissue TNF-alpha in skeletal muscle compared to both HF and C (Figure 1a–b). Mitochondrial enzyme activities were conversely higher in HFG than in control and HF animals, although this difference only reached statistical significance between HFG and control values (Figure 2a–b). Under the above conditions, ghrelin treatment was associated with normalization of the high-fat diet-induced skeletal muscle triglyceride accumulation (Figure 2c).

### Plasma TBARS, muscle redox state and muscle glutathione peroxidase activity (Figure 3)

**Figure 3:**
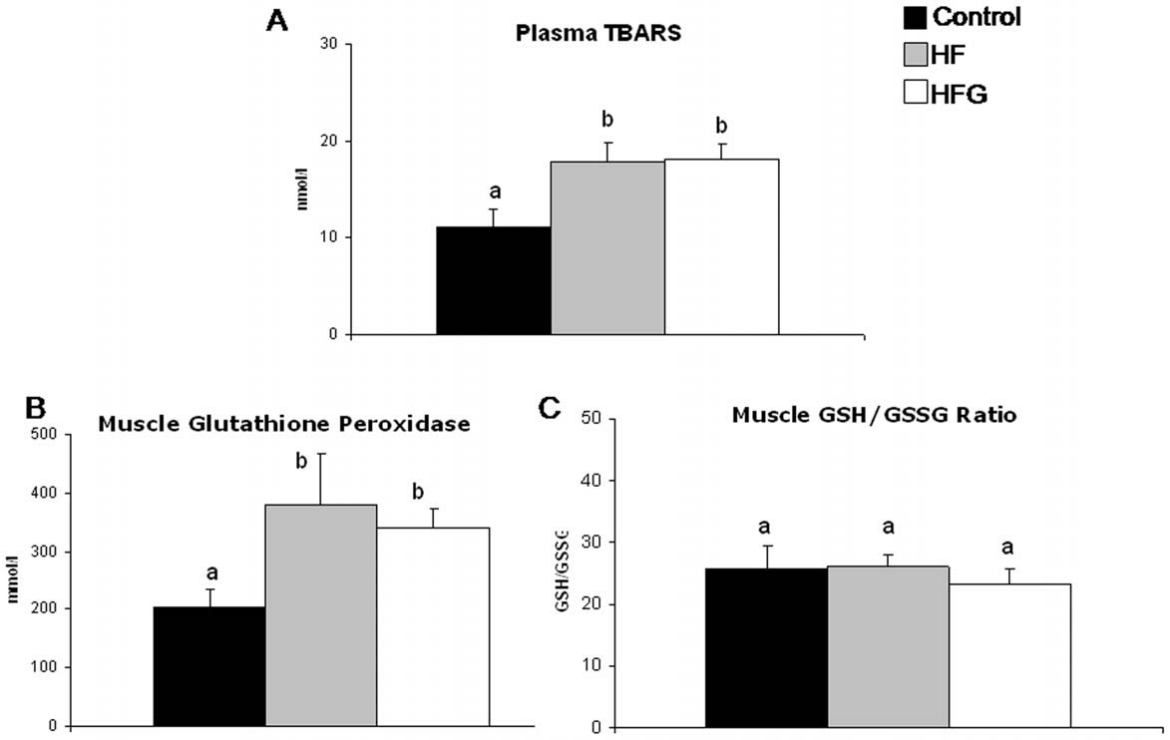
Acylated ghrelin effects are independent of changes in gastrocnemius muscle redox state. Effects of one-month high-fat feeding without (HF) or with (HFG) 4-day acylated ghrelin treatment on plasma TBARS concentration (a) and on gastrocnemius muscle glutathione peroxidase activity (b), and total 1 oxidized glutathione ratio (c). Different letters denote statistically significant differences (P<0.05 ANOVA and post-hoc test).

HF had high plasma TBARS concentrations indicating high systemic lipid peroxidation. Despite this alteration, skeletal muscle redox state as reflected by the GSSG/GSH ratio was unchanged in fat-fed animals. Glutathione peroxidase is a major antioxidant enzyme and we tested the hypothesis that lack of changes in muscle redox state is associated with a stimulation of its activity in HF. A stimulation of glutathione peroxidase activity was indeed observed in HF compared to control group. HFG had similar plasma TBARS, muscle GSSG/GSH ratio and glutathione peroxidase activity compared to the HF group.

### Muscle AKT, GSK and FOXO1 phosphorylation (Figure 4)

**Figure 4:**
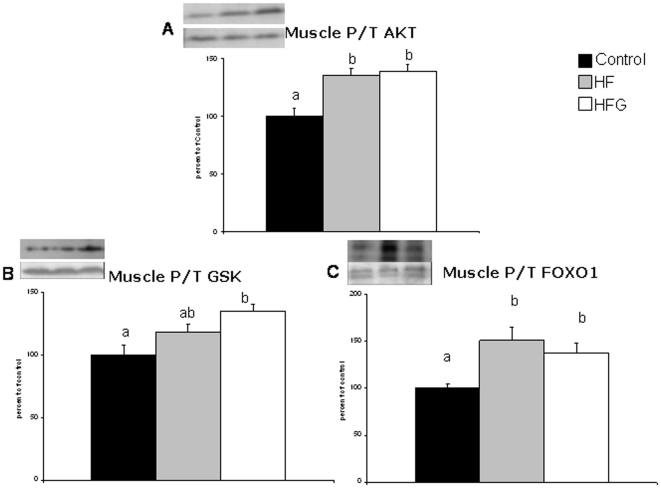
Acylated ghrelin during HF diet does not alter AKT-dependent gastrocnemius insulin signaling. Effects of one-month high-fat feeding without (HF) or with (HFG) 4-day acylated ghrelin treatment on gastrocnemius muscle phosphorylation of AKT (a), glycogen synthase kinase (GSK) (b) and FOXO1 (c). Different letters denote statistically significant differences (P<0.05 ANOVA and post-hoc test).

HF had high AKT phosphorylation compared to control animals, associated with higher phosphorylation of FOXO1 and GSK, although the latter did not reach statistical significance. A-Ghr had no independent impact on insulin signalling since muscle AKT, GSK and FOXO1 phosphorylation levels in HFG were comparable to HF, and higher than those observed in the control group.

## Discussion

The study demonstrated that acylated ghrelin administration following high-fat diet-induced weight gain induces anti-inflammatory changes and prevents triglyceride accumulation in rat skeletal muscle. These effects were associated with higher mitochondrial enzyme activities compared to control animals, and they were independent of changes in muscle redox state and of insulin signaling activation. Peripheral acylated ghrelin administration in fat-fed rats therefore results in a novel model of weight gain characterized by low inflammation, high mitochondrial enzyme activities and normal triglyceride content in skeletal muscle. Under the above conditions, ghrelin also had no negative impact on plasma metabolic profile but lead to a limitation of the diet-induced increase of circulating free fatty acids.

Reduction of muscle triglyceride content is a target for treatment of metabolic abnormalities that characterize weight gain and fat accumulation [1]. Acylated ghrelin treatment was reported to lower gastrocnemius muscle triglycerides in lean and uremic rodents [8], [9], and the current data are consistent with the concept that ghrelin prevents muscle lipid accumulation also in the presence of high dietary fat availability. Ghrelin effects on muscle triglycerides are clustered with enhanced tissue oxidative capacity compared to control animals, although this effect was not statistically significant when compared to high-fat fed animals treated with saline. Effects of acylated ghrelin towards enhanced muscle mitochondrial enzyme activities where previously demonstrated in lean and uremic animals, and more pronounced enhancement of muscle oxidative capacity could have potentially contributed to prevent triglyceride accumulation also in the current model [8], [9]. The current data also provide the first demonstration of antiinflammatory effects of ghrelin in skeletal muscle, consistent with previous reports in vitro and in vivo at systemic level [10], [12]. Since TNF-alpha may reduce skeletal muscle mitochondrial citrate synthase activity in vivo [3], low TNF-alpha could have contributed to ghrelin-induced mitochondrial changes compared to control animals. In addition to muscle changes, ghrelin had no negative impact on plasma metabolic profile in terms of glucose and insulin concentrations, and it limited the diet-induced increase in circulating free fatty acids. Taken together, the current findings indicate potentially beneficial muscle metabolic effects of sustained acylated ghrelin administration in fat-induced obesity. Notably, ghrelin effects were expectedly independent of reductions in body weight, that was instead further moderately increased during the four-day ghrelin administration. The latter change is in agreement with previous findings by us and others [7], [8], indicating that ghrelin may enhance body weight independently of enhanced food intake. This effect could theoretically involve changes in physical activity, that were not investigated under the current experimental design. Changes in body weight could have also at least in part involved ghrelin effects to enhance fat deposition in adipose tissue by stimulation of lipogenesis and potential inhibition of lipolysis [14], [15].

In the current study, one-month high-fat feeding was associated with enhanced rather than impaired phosphorylation of muscle AKT and further downstream steps of insulin signaling in the absence of acute insulin stimulation. This finding is in agreement with emerging evidence that insulin signaling activation under non-stimulated conditions characterizes relatively early stages of diet-induced obesity [2], and could favor the onset of obesity-associated metabolic complications [2]. Under the current experimental conditions, acylated ghrelin had no independent effects on AKT, GSK and FOXO1 phosphorylation compared to high-fat feeding per se. We recently demonstrated that acylated ghrelin treatment normalizes impaired AKT phosphorylation in gastrocnemius muscle in a rodent model of chronic kidney disease [9], but this effect appeared to be dependent of ghrelin-induced normalization of food intake in anorectic animals [9]. On the other hand, insulin-sensitizing effects of ghrelin did not occur in gastrocnemius muscle in lean rodents [13] and this finding is consistent with the current observations in diet-induced obesity.

Fat feeding per se did not modify muscle inflammation or mitochondrial enzyme activities, but the inability to lower tissue proinflammatory cytokines and to further enhance mitochondrial oxidative capacity likely contributed to muscle triglyceride accumulation in untreated fat-fed animals. Previous studies suggest that the reported effects of high-fat feeding on muscle inflammation and mitochondrial oxidative capacity are at least in part dependent on treatment duration. More prolonged treatments may result in higher inflammation and lower mitochondrial function, associated with the onset of oxidative stress [3], [16], while our findings agree with studies of comparable duration [17] in suggesting that adaptive mechanisms may limit fat-induced skeletal muscle alterations in early stages of dietary treatment. Enhanced glutathione peroxidase activity was indeed associated with high-fat feeding in the current model in agreement with previous reports [18], and it could have contributed to prevent changes in muscle redox state, inflammation and mitochondrial enzyme activities [4], [5]. Activation of muscle antioxidant defenses by fat feeding could have also masked potential independent antioxidant effects of acylated ghrelin, since such effects have been reported to involve glutathione availability in vitro [11], [12]. Also based on the above observations, the positive effects of ghrelin in the current model provide a strong rationale to further assess the impact of ghrelin administration in obesity models with different degrees of muscle metabolic abnormalities.

In conclusion, acylated ghrelin administration during high-fat feeding results in a novel model of weight gain with low inflammation and lower triglycerides in rat skeletal muscle, associated with high mitochondrial oxidative capacity compared to control animals. These effects are independent of changes in tissue redox state and insulin signaling, and they suggest a potential positive metabolic impact of acylated ghrelin in high-fat diet-induced weight gain.

## Materials and Methods

### Animals and experimental protocol

The experimental protocol was approved by the Committee for Animal Studies at Trieste University. Animals were housed in individual cages, and care was taken to minimize stress during all procedures, that did not involve invasive treatments and techniques. At the time of sacrifice adequate anaesthesia was achieved before any procedures were performed, as described below. Thirty 12-week-old male Wistar rats were purchased from Harlan-Italy (San Pietro al Natisone, Udine, Italy) and kept for two weeks in the Animal Facility of the University of Trieste in individual cages on a 12-h light/12-h dark cycle (0600 h/1800 h). Animals were then randomly assigned to undergo a high-fat feeding program for one month with a diet containing 60% energy from fat (HF, n = 20; diet composition: Protein 17%, Fat 34%, Carbohydrate approximately 45% with Fiber 5%) or a control diet containing 10% energy from fat (C, n = 10; Protein 23%, Fat 5%, Carbohydrate approximately 65% with Fiber 5%). Both diets were purchased from Mucedola, Settimo Milanese, MI, Italy. All animals were weighed and food intake was monitored two times per week. After thirty days, animals in the HF group were randomized to receive eight subcutaneous injections of either acylated rat ghrelin as previously reported [8], [13] (AnaSpec, San Jose, CA) (HFG, 200 µg ghrelin/injection) or saline, that was also administered to control rats. Injections were administered two times per day at 8 PM and 8 AM, beginning in the evening of day 1 with the last injection on the morning of day 4 before sacrifice. After the last injection, food was withdrawn for three hours before the intraperitoneal injection of an overdose of sodium pentobarbital (80 mg/kg). After achievement of adequate anaesthesia, gastrocnemius muscle was dissected and quickly frozen in liquid nitrogen to be stored at −80 before analyses. The epidydimal and retroperitoneal fat pads were then dissected and weighed, and blood was finally collected through cardiac puncture for plasma separation and storage at −80.

### Protein analysis

Total tissue protein was extracted from homogenized tissue as described [8], [13]. Nuclear protein extracts were obtained as follows: 100 mg tissue were homogenized in lysis buffer (10 mM HEPES pH 7,9, 10 mM KCl, 1,5 mM MgCl_2_, 1 mM DTT, 0,5 mM PMSF) containing protease inhibitors, followed by addition of 1% Nonidet P-40. The homogenate was incubated on ice for 20 min and then centrifuged at 12500 rpm 4°C for 30 seconds. Pellets were resuspended in 100 µl extraction buffer (20 mM HEPES pH 7,9, 420 mM NaCl, 1,5 mM MgCl_2_, 0,2 mM EDTA, 5% Glycerol, 1 mM DTT, 0,5 mM PMSF) added with protease inhibitors and the tube was gently rocked on ice for 30 min. The mixture was then spun at 12500 rpm at 4°C for 15 min and the resulting supernatant containing nuclear proteins was stored at −80 C until analyses. Protein concentration in all samples was measured by spectrophotometer (BCA Protein Assay Reagent, Pierce, Rockford, IL, USA). For measurement of tissue p65 NFkB subunit (TransAM, Active Motif North America, Carlsbad, CA, USA) and TNF-alpha (Pierce Biotechnology, Rockford, IL, USA) nuclear and total tissue proteins respectively and commercially available kits were used. Total tissue proteins and commercially available antibodies (Cell Signaling Technology, Danvers, Ma, USA) were also used to measure phosphorylated and total protein levels of AKT and its downstream targets glycogen synthase kinase (GSK) and FOXO1 by immunoblotting.

### Cytochrome c oxidase (COX) and citrate synthase (CS) activity

COX and CS enzyme activities were measured spectrophotometrically from tissue homogenates as referenced [8].

### Muscle total and oxidized glutathione, glutathione peroxidase activity

Glutathione is the major antioxidant system in skeletal muscle and tissue total and oxidized glutathione were therefore determined as indicators of redox state. Muscle total and oxidized glutathione were measured using the HT Glutathione Assay Kit (Trevigen, Gaithersburg, MD, USA) according to manufacturer's instructions. Briefly, ∼50 mg of gastrocnemius sample were cleaned from blood and fibrous residues and homogenized in ice cold 5% (w/v) metaphosphoric acid (20 ml/g tissue). Homogenates were then centrifuged (12.000×g for 15′) and the supernatant was used for total glutathione measurement after appropriate dilution. For oxidized glutathione, samples were pretreated with 2 M 4-vinylpyridine. Reduced glutathione was calculated by subtracting the oxidized fraction from the total. To further determine potential diet- and ghrelin-induced muscle changes in enzyme activity of the antioxidant scavenging enzyme glutathione peroxidase, the latter was also determined by spectrophotometry and a commercially available kit following the manufacturer's instructions (HT Glutathione Peroxidase Assay Kit, Trevigen, Gaithersburg, MD, USA).

### Plasma metabolic profile

Plasma insulin concentration was measured by ELISA using a commercially available kit (Insulin Rat/Mouse ELISA, DRG Diagnostics, Marburg, Germany Marburg). Plasma FFA were determined spectrophotometrically using an acyl-CoA oxidase-based colorimetric kit (NEFA-C, WAKO Pure Chemical Industries). Plasma thiobarbituric acid-reactive substance (TBARS) were measured spectrophotometrically using commercial kit (TBARS assay kit, OXItek Zeptometrix Corporation).

### Statistical analysis

Results in the three groups were compared using One-Way ANOVA. Post-hoc tests were then used to compare results between two groups. P values<0.05 were considered statistically significant.
